# Construction and validation of a nomogram based on inflammatory, nutritional and metabolic indicators for predicting the risk of papillary thyroid carcinoma

**DOI:** 10.3389/fonc.2026.1917420

**Published:** 2026-10-07

**Authors:** Ziyu Chen, Hang Su, Hongfeng Wu, Quancang Men, Xinning Lv, Manli Zhang, Fenghua Zhang

**Affiliations:** 1 Department of Thyroid and Breast Surgery, Hebei General Hospital, Shijiazhuang, China; 2 General Affairs Office, Hebei General Hospital, Shijiazhuang, China; 3 Department of Gynecology, Hebei General Hospital, Shijiazhuang, China; 4 Department of Thyroid and Breast Surgery, Cangzhou People’s Hospital, Cangzhou, China

**Keywords:** Hashimoto’s thyroiditis, metabolic syndrome, nomogram, papillary thyroid carcinoma, prognostic nutritional index, uric acid

## Abstract

**Objective:**

To analyze the correlation between inflammation, nutrition and metabolic indicators and the risk of papillary thyroid carcinoma (PTC), and to construct and validate a nomogram for predicting PTC risk among patients with thyroid nodules undergoing surgery, to assist clinicians in making more individualized clinical decisions.

**Study design:**

Retrospective population-based cohort study.

**Methods:**

A retrospective study was conducted on the clinical medical records of patients with thyroid masses who underwent surgical treatment at Hebei General Hospital from 2020 to 2024. Independent risk factors for the onset of PTC were screened through Logistic regression analysis. A risk prediction model for the onset of PTC based on inflammatory, nutritional and metabolic indicators was constructed using R4.3.3 software and externally validated.

**Results:**

A total of 1727 patients who underwent thyroidectomy for thyroid nodules were enrolled, among whom 1167 patients (67.6%) were pathologically-confirmed papillary thyroid carcinoma (PTC), and 560 patients (32.4%) had benign thyroid nodules after surgery. Multivariate logistic regression analysis demonstrated combined Hashimoto’s thyroiditis (HT) (OR = 1.887, 95%CI: 1.174-3.031, P = 0.009), combined metabolic syndrome (MetS) (OR = 2.157, 95%CI: 1.449-3.211, P < 0.001), and elevated uric acid (UA) (OR = 1.640, 95%CI: 1.112-2.417, P = 0.013) were independent risk factors for PTC onset. Age ≥ 55 years (OR = 0.318, 95%CI: 0.238-0.424, P < 0.001), other marital status (OR = 0.067, 95%CI: 0.012-0.392, P = 0.003), and increased prognostic nutritional index (PNI) (OR = 0.934, 95%CI: 0.902-0.966, P < 0.001) were independent protective factors against PTC onset. Based on the above independent risk factors, a nomogram for PTC was constructed. The AUC of the ROC curve in the modeling group was 0.702, and that in the external validation group was 0.721, indicating that the nomogram has good predictive performance. The calibration curve and decision analysis curve also indicate that the nomogram has good predictive performance and clinical application efficacy.

**Conclusion:**

Combined HT, combined MetS, and elevated UA are independent risk factors for PTC onset, whereas age ≥ 55 years, other marital status, and PNI level represent independent protective factors.

## 1 Introduction

Thyroid cancer is the most common malignant tumor in the endocrine system. Its global incidence rate has shown a significant and rapid upward trend over the past few decades and has become a health issue of great concern worldwide (1). Among the various pathological types of thyroid cancer, papillary thyroid carcinoma (PTC) accounts for the vast majority (approximately 85%-90%) (2). Fortunately, PTC usually progresses slowly and has a good prognosis, with a 10-year survival rate of over 90% (3). However, some PTC patients still exhibit aggressive biological behaviors, such as lymph node metastasis and extrathyroidal invasion, which are closely related to poor prognosis (4). Therefore, in-depth exploration of the driving factors for the occurrence and development of PTC and early identification of high-risk patients with invasive clinicopathological features are of vital significance for achieving individualized and precise treatment and improving the prognosis of patients.

Traditionally, the main risk factors for PTC include exposure to ionizing radiation during childhood, genetic susceptibility, and family history, etc (5).These factors can only explain a small proportion of cases. The rapid increase in the incidence rate suggests that environmental factors and the internal factors of the host may play a key role. In recent years, the role of the tumor microenvironment in the occurrence and development of tumors has received increasing attention. TME is a complex ecosystem, in which a persistent chronic inflammatory state is regarded as an important biological feature promoting tumorigenesis (6). Systemic inflammatory responses can promote cell proliferation, angiogenesis and inhibit apoptosis by releasing various cytokines and growth factors, thereby creating a microenvironment conducive to tumor occurrence and progression (7). In addition, the nutritional and metabolic status of the body is also closely related to the occurrence and development of tumors. Malnutrition or cachexia can lead to a decline in immune function and weaken the body’s anti-tumor ability (8). In addition to inflammatory, nutritional and metabolic biomarkers, other readily available clinical indicators such as ABO blood type have also been applied to risk stratification research of PTC. ABO antigens can influence the biological behavior of tumors through inflammatory regulation, immune surveillance, and interaction with the tumor microenvironment (9). However, the existing research evidence on this association is inconsistent. This retrospective cohort lacks data related to ABO blood type and Rh factor, therefore such variables were not incorporated into the proposed prediction model.

Although several nomograms for predicting PTC risk have been reported, most existing models rely heavily on ultrasound imaging or genetic markers, and few simultaneously integrate systemic inflammatory, nutritional, and metabolic indicators. For patients with incomplete or ambiguous imaging data, our model can provide supplementary preoperative risk stratification. Its incremental value consists in integrating multiple panels of systemic biomarkers to fill the clinical gap in scenarios where high-quality ultrasound parameters are inaccessible, serving patients scheduled for thyroid nodule surgery.

## 2 Methods

### 2.1 Patient selection

This study retrospectively analyzed the clinical case data of patients with thyroid masses who were treated with surgery at Hebei General Hospital from January 1, 2020 to December 31, 2024. We used the following inclusion criteria to select eligible patients: 1) Age ≥18 years; 2) Postoperative paraffin pathology confirmed PTC or benign thyroid mass. 3) No other malignant tumors were combined; 4) The data on demographic and clinicopathological characteristics are complete. The study was approved by the Medical Ethics Committee of the Hebei General Hospital (NO. 2025-LW-0238) in accordance with the Declaration of Helsinki.

### 2.2 Data collection

The variables extracted from the selected cohort were as follows: Age, gender, marital status, whether combined with Hashimoto’s thyroiditis (HT), systemic immune inflammatory index (SII), systemic inflammatory response index (SIRI), prognostic nutritional index (PNI), total protein, albumin, whether combined with metabolic syndrome (MetS), whether elevated uric acid (UA), D-dimer, mean platelet volume (MPV) and platelets Distribution width (PDW).

### 2.3 Statistical analysis

In this study, SPSS 26.0 software was used for basic data processing and statistical analysis, and R4.3.3 software was utilized for the construction and verification of the nomogram. After the normality test of the measurement data, those conforming to the normal distribution were described as mean ± standard deviation (x ± s), and the independent sample t-test was used for comparison between groups. Those with non-normal distribution were described as median and quartile [M (P25, P75)], and the Mann-Whitney U test was used for comparison between groups. Counting data were described as the number of cases [n (%)], and chi-square test was used for comparison between groups. The independent risk factors for the onset of PTC were screened through Logistic regression analysis. Univariate logistic regression was first performed for all candidate variables. Variables with P <0.05 in univariate analyses were then entered into multivariate logistic regression using the enter (forced-in) method, rather than stepwise selection, to avoid bias and overfitting introduced by stepwise algorithms. A Logistic regression model based on significant independent risk factors was established using the rms software package of R software (version 4.3.3), and visualized using a nomogram. Multicollinearity diagnosis was performed before modeling. Take the variance inflation factor (VIF) < 10 as the standard. The predictive performance of the prediction model was evaluated by drawing the receiver operating characteristic (ROC) curve and calculating the area under the curve (AUC). The consistency between the predicted probability of the prediction model and the actual observed probability is evaluated by drawing a calibration curve. In addition, decision curve analysis (DCA) was applied to further evaluate the clinical application efficacy of the nomogram.

## 3 Result

### 3.1 Comparison of clinical characteristics between the PTC group and the benign thyroid nodule group

From January 2020 to December 2024, a total of 1727 patients who underwent surgical resection for thyroid nodules and met the inclusion criteria were enrolled. Among them, 1167 (67.6%) patients were allocated to Group I (PTC) and 560 (32.4%) patients to Group II (benign thyroid nodules). Chi-square test was used to compare clinical data between Group I and Group II. Statistically significant inter-group differences (P<0.001) were found in age, marital status, coexisting Hashimoto’s thyroiditis (HT), coexisting metabolic syndrome (MS), and elevated uric acid (UA). For laboratory parameters, the Mann-Whitney U test and independent-samples t-test revealed significant differences in prognostic nutritional index (PNI) (P = 0.006) and D-dimer (P<0.001) between the two groups. However, no statistically significant differences were observed in systemic immune-inflammation index (SII), systemic inflammation response index (SIRI), mean platelet volume (MPV), platelet distribution width (PDW), total protein, or other indicators (all P>0.05), as shown in **Table 1**.

**Table 1:** Comparison of clinical characteristics between the PTC group and benign thyroid nodule group.

Variable	Group I (n=1167)	Group II (n=560)	*X^2^/Z/t*	*P*
Age			103.240	**<0.001**
<55years	819(70.2%)	251(44.8%)		
≥55years	348(29.8%)	309(55.2%)		
Gender			3.178	0.075
Female	869(74.5%)	439(78.4%)		
Male	298(25.5%)	121(21.6%)		
Marital Status			29.989	**<0.001**
Single	52(4.5%)	5(0.9%)		
Married	1098(94%)	529(94.5%)		
Other	17(1.5%)	26(4.6%)		
With HT			15.581	**<0.001**
No	1000(85.7%)	517(92.3%)		
Yes	167(14.3%)	43(7.7%)		
SII	546.06 (395.62, 743.48)	500.47 (405.88, 775.80)	-1.126	0.260
SIRI	0.54(0.38, 0.75)	0.54(0.38, 0.86)	-1.500	0.134
PNI	52.85(50.15, 55.30)	53.02(50.65, 55.98)	-2.775	**0.006**
Total Protein(g/L)	74.00 ± 5.36	73.66 ± 5.37	-1.251	0.211
With MetS			24.021	**<0.001**
No	913(78.2%)	493(88.0%)		
Yes	254(21.8%)	67(12.0%)		
Elevated UA			14.596	**<0.001**
No	929(79.6%)	488(87.1%)		
Yes	238(20.4%)	72(12.9%)		
D-dimer(Mg/L)	0.24(0.16, 0.36)	0.29(0.18, 0.43)	-4.646	**<0.001**
MPV(fL)	10.20(9.70, 10.90)	10.20(9.60, 10.80)	-1.157	0.247
PDW(fL)	11.50(10.50, 13.00)	11.40(10.30, 12.60)	-1.703	0.089

Bold values represent results with statistically significant differences (p<0.05). Ref., reference grop in logistic regression analysis.

### 3.2 Logistic regression analysis of factors influencing the risk of PTC onset

In the modeling group, univariate Logistic regression analysis was conducted with age, gender, marital status, whether combined with HT, SII, SIRI, PNI, total protein, albumin, whether combined with MetS, whether elevated uric acid, D-dimer, MPV and PDW as independent variables, and whether PTC occurred as dependent variable. The results showed that age, marital status, combined HT, PNI, combined MetS, and elevated uric acid were significantly associated with the onset of PTC. However, gender, SII, SIRI, total protein, D-dimer, MPV and PDW were not related to the onset of PTC. The six statistically significant variables screened out by univariate Logistic regression analysis: age, marital status, whether combined with HT, PNI, whether combined with MetS, and whether elevated uric acid were included in the multivariate Logistic regression analysis. The results show that age, marital status, combined HT, PNI, combined MetS, and elevated uric acid are independent risk factors affecting the onset of PTC, as shown in **Table 2**.

**Table 2:** Logistic regression analysis of the risk of PTC in the modeling group.

Variable	Univariate analysis			P	Multivariate analysis			P
	B	OR	95%CI		B	OR	95%CI	
Age								
<55years				**Ref.**				**Ref.**
≥55years	-1.088	0.337	0.257~0.442	**<0.001**	1.146	0.318	0.238~0.424	**<0.001**
Gender								
Female				Ref.				
Male	0.012	1.012	0.742~1.379	0.941				
Marital status								
Single				**Ref.**				**0.005**
Married	-1.824	0.161	0.038~0.690	**0.014**	-1.348	0.260	0.059~1.143	**0.074**
Other	-3.285	0.037	0.007~0.204	**<0.001**	-2.699	0.067	0.012~0.392	**0.003**
With HT								
No				**Ref.**				**Ref.**
Yes	0.687	1.987	1.273~3.102	**0.002**	0.635	1.887	1.174~3.031	**0.009**
SII	0.000	1.000	0.999~1.000	0.153				
SIRI	-0.180	0.835	0.638~1.095	0.192				
PNI	-0.067	0.935	0.906~0.965	**<0.001**	-0.069	0.934	0.902~0.966	**<0.001**
Total Protein(g/L)	0.061	1.062	1.017~1.109	**0.006**				
With MetS								
No				**Ref.**				**Ref.**
Yes	0.472	1.603	1.113~2.309	**0.011**	0.769	2.157	1.449~3.211	**<0.001**
Elevated UA								
No				**Ref.**				**Ref.**
Yes	0.478	1.613	1.124~2.315	**0.010**	0.494	1.640	1.112~2.417	**0.013**
D-dimer(mg/L)	-0.155	0.857	0.702~1.046	0.129				
MPV(fL)	0.029	1.030	0.947~1.120	0.494				
PDW(fL)	0.053	1.054	0.989~1.124	0.106				

Bold values represent results with statistically significant differences (p<0.05). Ref., reference grop in logistic regression analysis.

### 3.3 Construct and validate the nomogram of PTC risk based on inflammatory, nutritional and metabolic indicators

Based on the results of multivariate logistic regression analysis, six independent risk factors, namely age, marital status, combined HT, PNI, combined MetS, and elevated uric acid, were finally extracted. The rms software package of R software (version 4.3.3) was used to establish a risk prediction model for the onset of PTC based on inflammatory, nutritional and metabolic indicators. And visualize it using a nomogram (**Figure 1**). The AUC of the modeling group was 0.702, and that of the external validation group was 0.721, indicating that the nomogram performed well in predicting the risk of PTC, as shown in **Figures 2**, **3**. Secondly, a calibration curve was further plotted. The results showed that the prediction results of the prediction model had a high degree of fit with the actual observed results, indicating that the prediction model had a good consistency with the ideal model, as shown in **Figures 4**, **5**. Finally, we conducted a decision curve analysis. The nomogram demonstrated excellent clinical application efficacy, as shown in **Figure 6** and **Supplementary Figure 1**.

**Figure 1:**
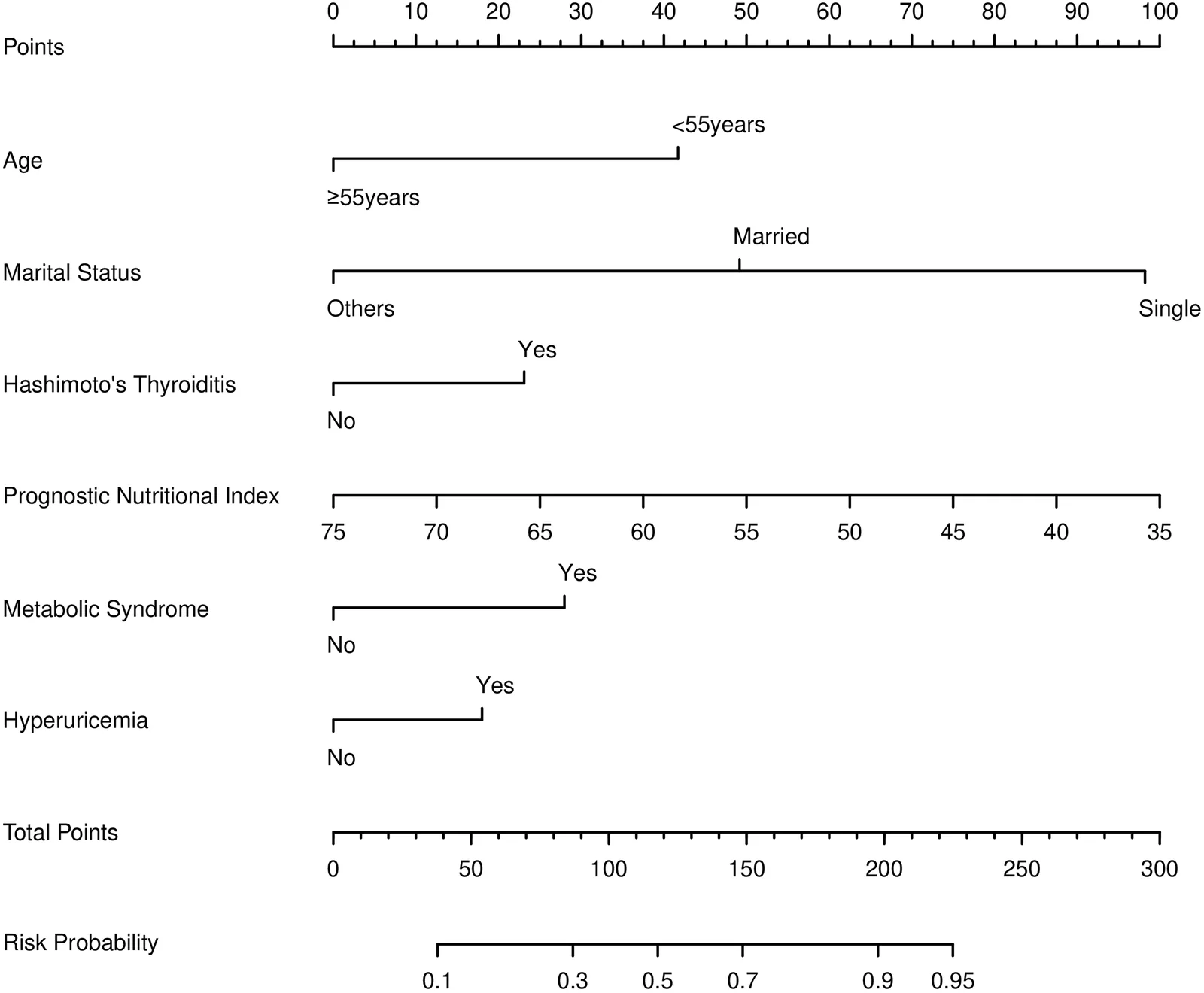
The nomogram for predicting the risk of papillary thyroid carcinoma.

**Figure 2:**
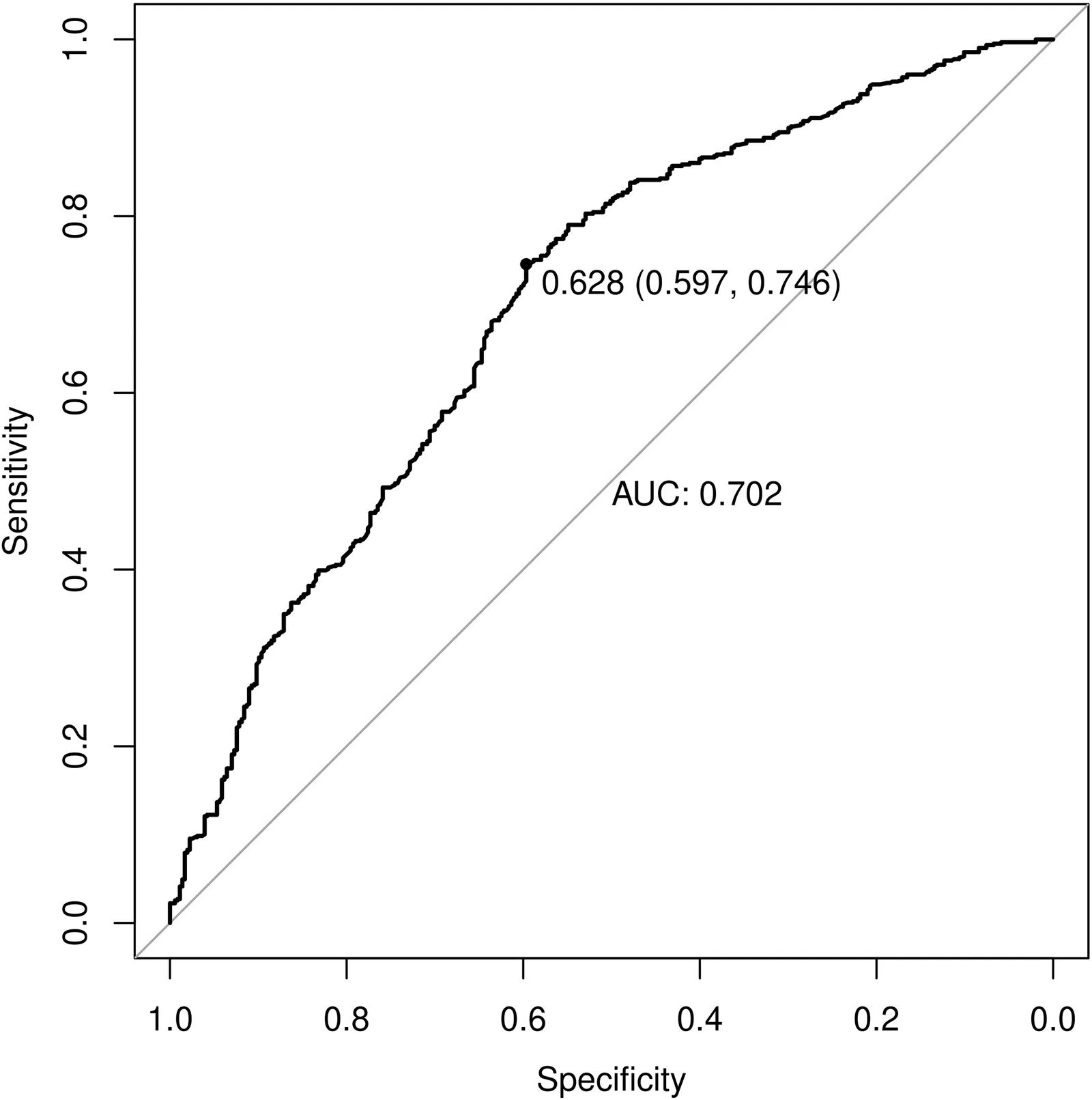
The ROC curve of the risk nomogram for papillary thyroid carcinoma in the training cohort.

**Figure 3:**
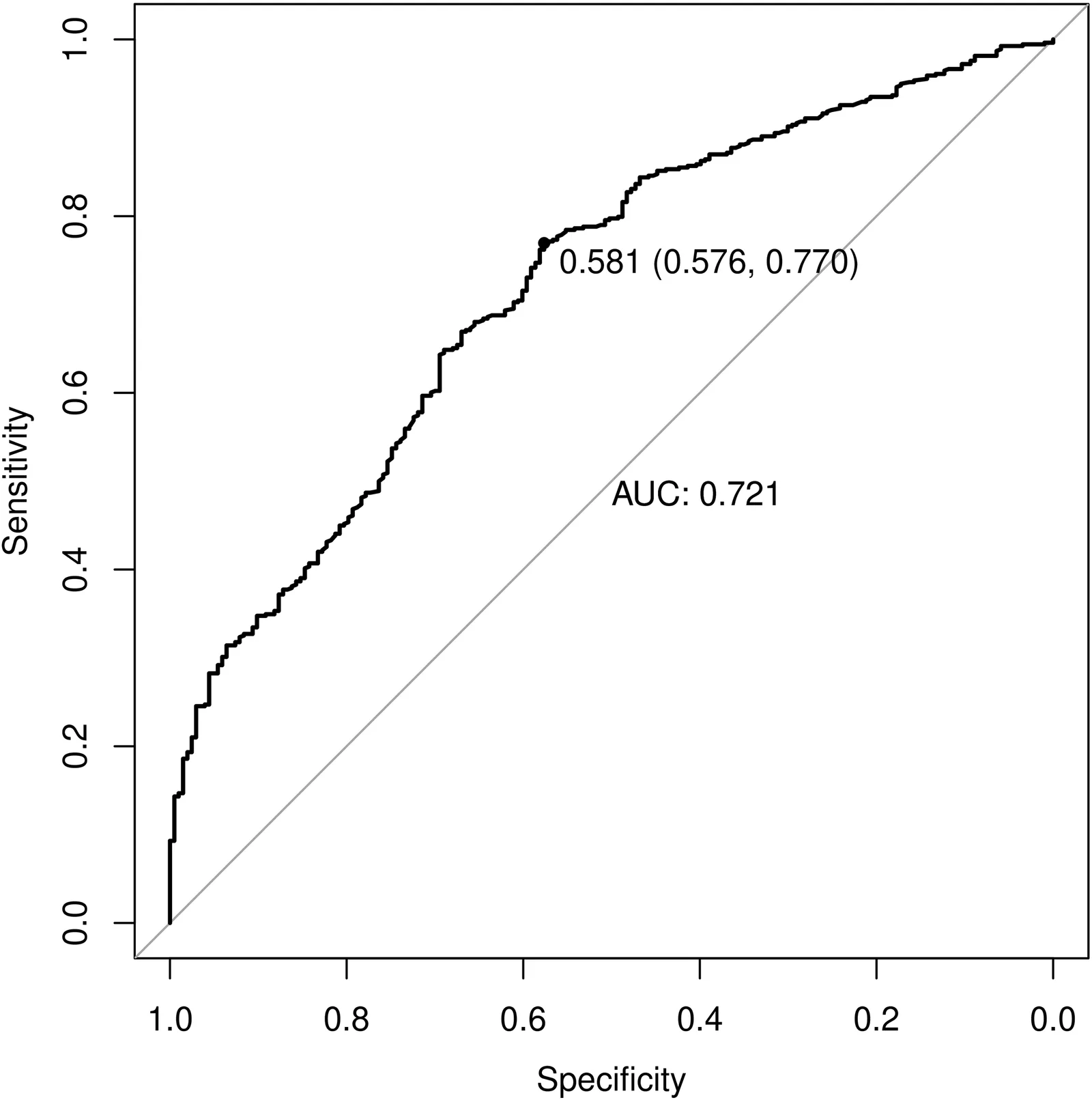
The ROC curve of the risk nomogram for papillary thyroid carcinoma in the validation cohort.

**Figure 4:**
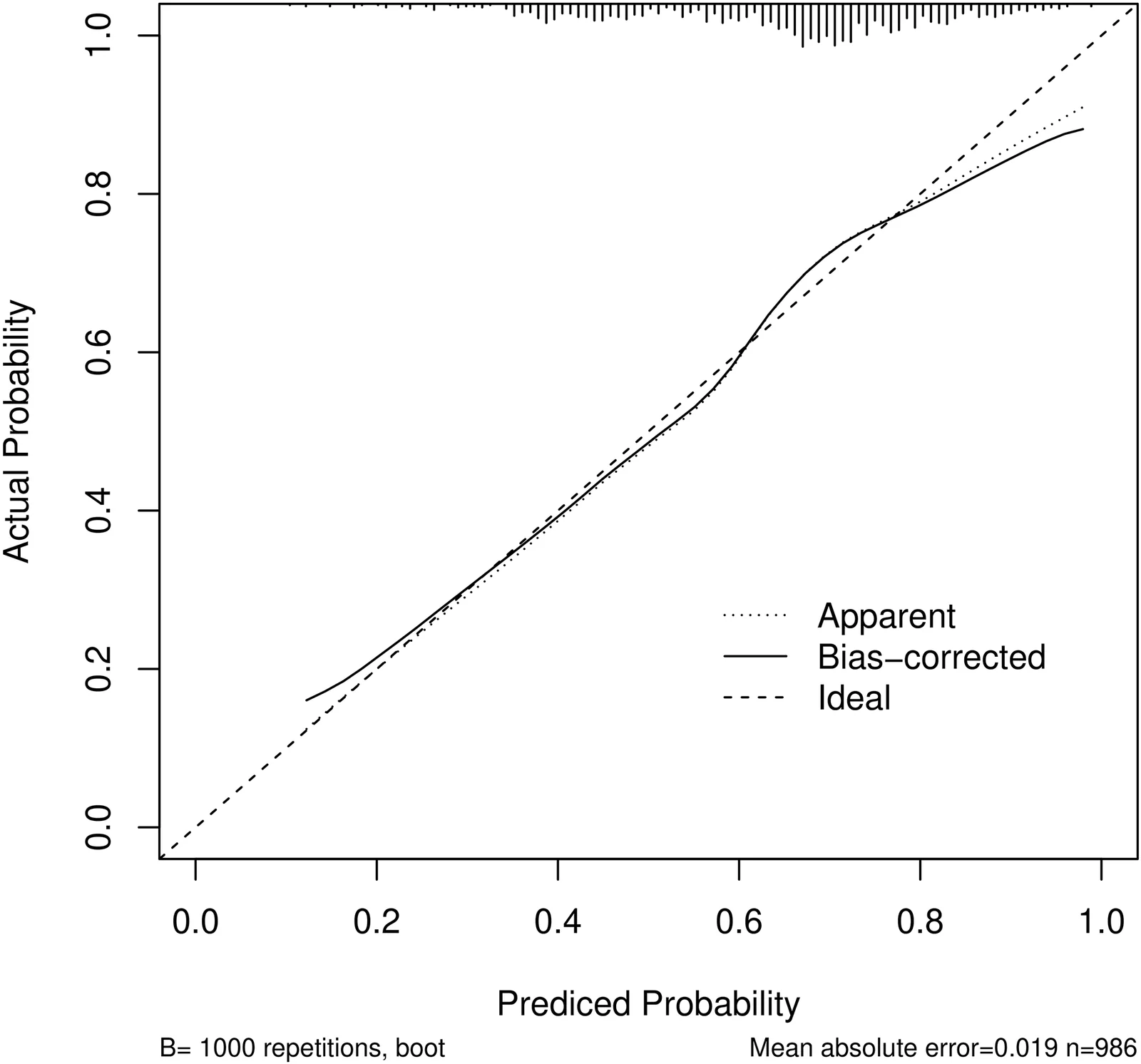
The calibration curve of the risk nomogram for papillary thyroid carcinoma in the training cohort.

**Figure 5:**
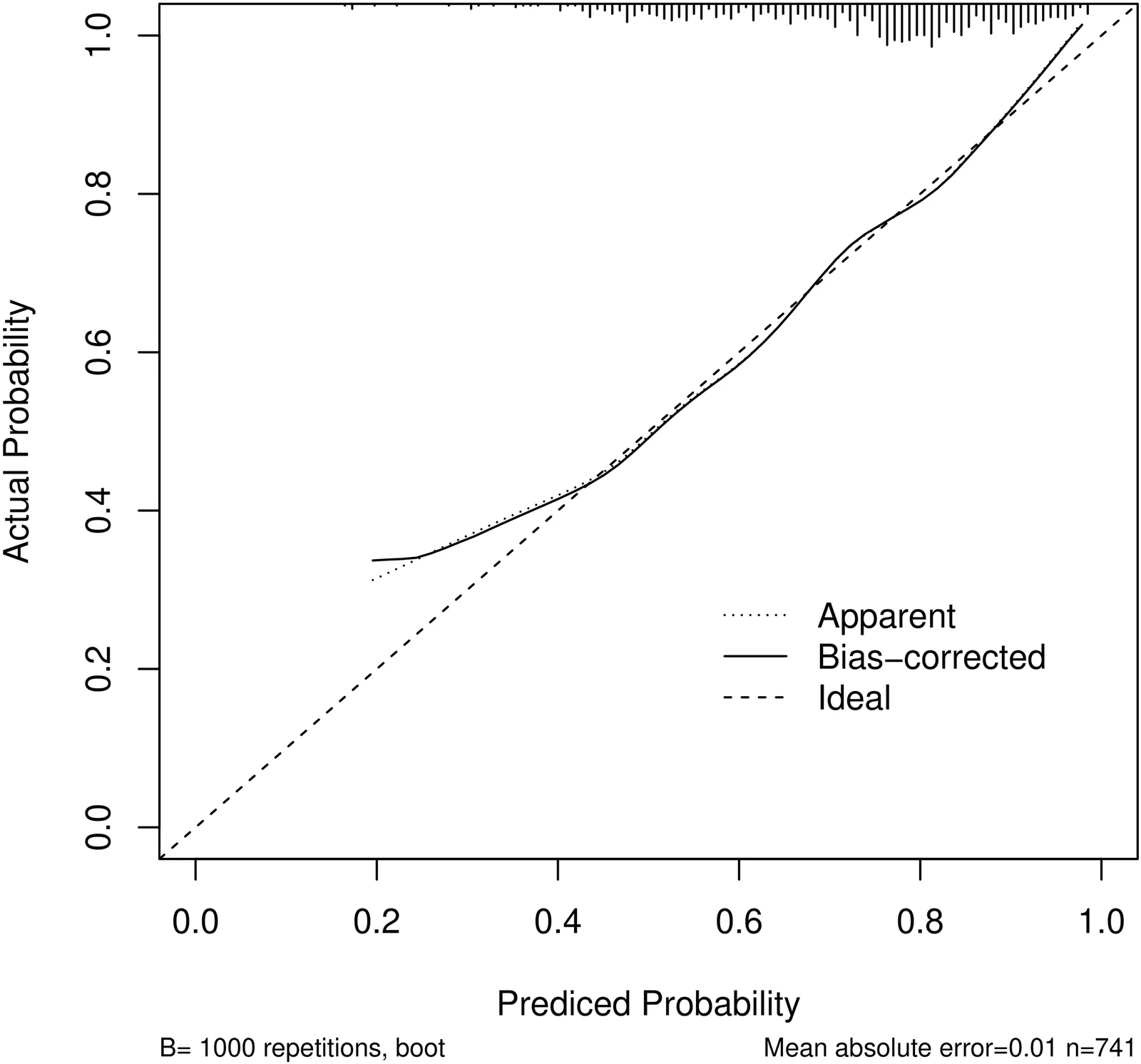
The calibration curve of the risk nomogram for papillary thyroid carcinoma in the validation cohort.

**Figure 6:**
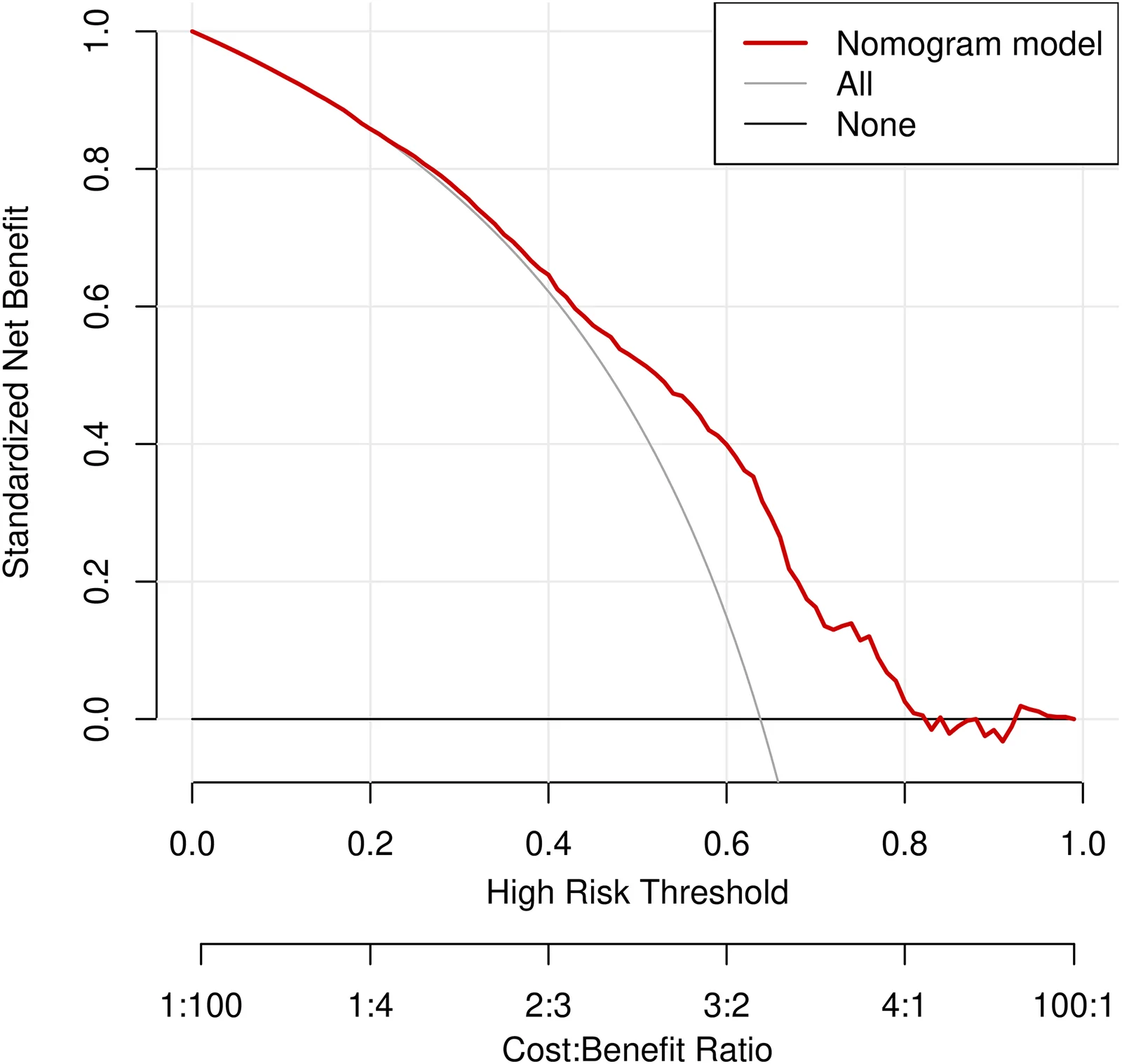
The DCA curve of the risk nomogram for papillary thyroid carcinoma in the validation cohort.

## 4 Discussion

### 4.1 Correlation between HT and PTC risk

PTC is one of the fastest-growing malignancies globally. Although its overall prognosis is favorable, some patients still exhibit aggressive behaviors such as lymph node metastasis and extrathyroidal extension (2, 3). Therefore, identifying its risk factors is crucial for early prevention and precise diagnosis and treatment.

HT is a common clinical autoimmune disease, also known as chronic lymphocytic or autoimmune thyroiditis, typically characterized by goiter, lymphocytic infiltration, and increased autoimmune antibodies (10). In 1955, Dailey first investigated the relationship between HT and PTC (11). In recent years, numerous studies have shown that chronic inflammation is an indispensable participant in cancer development (12–14). Currently, most studies confirm a positive correlation between HT and TC incidence, and multiple large-scale epidemiological studies have further validated this association. A systematic review and meta-analysis by Abbasgholizadeh P et al. (15), which included 50 studies (27 included in quantitative synthesis), found that the overall risk of thyroid malignancy in HT patients was 1.36 times that of the normal population (OR: 1.36, 95%CI: 1.05–1.77). Specifically, the risk of PTC was 1.65 times (95%CI: 1.04–2.61), the risk of medullary thyroid carcinoma (MTC) was 2.70 times (95%CI: 1.20–6.07), and the risk of thyroid lymphoma was 12.92 times (95%CI: 2.15–77.63) higher in HT patients compared to the normal population, with no significant association with anaplastic or follicular carcinoma. In 2023, a retrospective cohort study by Yang Y et al. (16) including 632 TC patients found that coexisting HT increased the risk of PTC by 88.7% (OR = 1.887). Patients with coexisting HT were younger, had a higher proportion of females (88.3% vs. 73.1%), higher levels of TSH, TPOAb, and TGAb (P<0.001), and smaller tumor diameters (P = 0.034). The biological mechanisms underlying this association are multi-layered and complex. Although there is no direct evidence that HT directly causes PTC, the chronic inflammatory microenvironment created by HT is considered a significant promoter of PTC development and progression. Of note, most prior studies focused on general populations, while our observation was derived exclusively from surgical patients with thyroid nodules. Whether this association persists in non-surgical thyroid-nodule populations requires further validation. Future research should further elucidate the complex interaction between these two diseases and provide more personalized treatment plans for PTC patients.

### 4.2 Correlation between PNI and PTC risk

Nutritional status is a crucial factor influencing tumor occurrence and progression. Good nutritional status can reduce tumor risk by maintaining immune function and regulating metabolic pathways (17). PNI is a comprehensive nutritional assessment index based on serum albumin levels and peripheral blood lymphocyte count. Initially used to assess preoperative nutritional status and surgical risk, it has been widely applied in predicting tumor risk and prognosis in recent years (18). The formula for PNI is: serum albumin (g/L) + 5 × peripheral blood lymphocyte count (×10^9^/L).

A retrospective study by Qu Z et al. (19) on 215 hepatocellular carcinoma (HCC) patients undergoing radical resection found that PNI was an independent prognostic factor for overall survival (OS) and recurrence-free survival (RFS). Using a cut-off of 43.75, patients with high PNI (>43.75) had significantly better prognosis than those with low PNI (≤43.75), suggesting a synergistic anti-tumor effect of good nutritional and immune status. A retrospective study including 511 colorectal cancer (CRC) patients found that preoperative PNI was an independent prognostic factor for OS and progression-free survival (PFS). Using a cut-off of 48.65, patients with low PNI (≤48.65) had significantly worse OS and PFS than those with high PNI (>48.65), and multivariate analysis confirmed preoperative PNI as an independent predictor of OS and PFS in CRC patients (20). The results of this study show that PNI is an independent protective factor for PTC (OR = 0.934), meaning that for each unit increase in PNI, the risk of disease decreases by approximately 6.6%. Reports on the correlation between PNI and PTC risk are relatively scarce domestically and internationally. However, it is clear that PNI cleverly integrates the body’s nutritional reserves and immune status, and its protective effect stems from their synergistic action. A high PNI value indicates that the patient is simultaneously in an ideal state of “good nutrition” and “intact immunity.” This builds two lines of defense for the body: good nutrition ensures normal cellular function and genomic stability, while intact lymphocyte function provides powerful immune surveillance and clearance capabilities. The results of this study further validate the reliability of PNI as an independent protective factor for PTC, providing a new reference for early clinical identification of high-risk PTC populations. Notably, PNI had an OR of 0.934, representing a modest per-unit effect: each one-unit PNI increase was associated with a 6.6% relative reduction in PTC odds. Despite statistical significance, PNI acts only as a moderate modifying factor instead of a strong dominant predictor. Meaningful risk differences occur across broad PNI ranges rather than single-unit changes, and no validated P-specific cutoff exists for PTC risk stratification. Compared with HT (OR = 1.887) and MetS (OR = 2.157), PNI shows weaker individual predictive power. Even so, this low-cost, routinely measured composite biomarker still provides incremental predictive value when combined with inflammatory-metabolic variables.

### 4.3 Correlation between MetS and PTC risk

Metabolic disorders increase cancer-related morbidity and mortality (21). MetS is not a single disease but a comprehensive metabolic disorder syndrome. Clinical diagnosis requires meeting ≥3 of the following five criteria: central obesity, hypertriglyceridemia, low HDL-C, hypertension, and elevated fasting glucose (22). The results of this study show that MetS is an independent risk factor for PTC (OR = 2.157), meaning that individuals with MetS have more than twice the risk of developing PTC compared to those without. A nationwide population-based cohort study from South Korea showed that MS significantly increased the risk of TC by 15% (HR = 1.15, 95%CI: 1.13–1.17). This association was significant only in the obese population (HR = 1.10, 95%CI: 1.07–1.13) and not in the non-obese population (HR = 1.002, 95%CI: 0.98–1.03), and it was more pronounced in males (23). A 2023 cohort study by Kim J et al. (24) based on a young Korean population showed a significant dose-response relationship between cumulative exposure to MetS and TC risk: HRs for 1–4 exposures were 1.12 (1.02–1.23), 1.25 (1.10–1.42), 1.33 (1.15–1.55), and 1.48 (1.25–1.75), respectively (trend P<0.01), with the association being more prominent in males and non-obese individuals.

Current research suggests that insulin resistance (IR) is the cornerstone of MetS, referring to decreased sensitivity to insulin, leading to compensatory hyperinsulinemia. Additionally, visceral fat, acting as an endocrine organ, increases pro-inflammatory cytokine secretion and decreases adiponectin under conditions of hypoxia and macrophage infiltration, driving the chronic inflammation-IR network of MS (25). Furthermore, elevated leptin levels can simultaneously activate the PI3K/Akt and MAPK/ERK pathways via LepR and cross-communicate with thyroid hormone synthesis signals along the hypothalamic-pituitary-thyroid axis, dual-driving cell proliferation (26). Conversely, in obesity and MetS states, levels of adiponectin, which has anti-inflammatory, anti-proliferative, and insulin-sensitizing properties, are significantly reduced, losing activation of the AMPK-p21 axis, thereby removing the “brake” on cell growth and synergistically promoting tumor proliferation and inflammation progression (27). It should be highlighted that our result was observed among surgical thyroid-nodule patients. Whether metabolic intervention could modify PTC risk remains unclear and deserves further investigation.

### 4.4 Correlation between elevated uric acid and PTC risk

An increase in uric acid is a typical manifestation of impaired purine metabolism, traditionally considered a risk factor for gouty arthritis and kidney stones. Its role in tumor development has gained increasing attention in recent years. Uric acid is not only a marker of metabolic disturbance but also possesses biological characteristics such as pro-oxidative stress and pro-inflammatory effects, which can participate in tumor occurrence and progression through various pathways (28). Currently, accumulating studies suggest that serum uric acid levels are associated with the occurrence of various cancers. In 2019, a meta-analysis including 32 studies found that hyperuricemia was associated with an increased risk of overall cancer mortality (RR = 1.15, 95%CI: 1.05–1.26), with higher risk in women (RR = 1.26, 95%CI: 1.09–1.45); for each 1 mg/dL increase in uric acid, the overall cancer risk increased slightly (RR = 1.01, 95%CI: 1.01–1.01) (29). Additionally, a study found a “J”-shaped relationship between uric acid levels and breast cancer risk, with a detection threshold of 3.6 mg/dL (18). Currently, there are few domestic and international studies on UA and PTC. In our multivariate model, elevated uric acid independently increased PTC risk (OR = 1.640, 95%CI:1.112-2.417, P = 0.013). Mechanistically, excess urate induces reactive-oxygen-species overproduction and DNA damage; it also activates the NLRP3 inflammasome to release pro-inflammatory cytokines, creating a pro-tumor microenvironment (30–32). Additionally, HUA interacts with insulin resistance and may amplify the carcinogenic effect of metabolic syndrome (33).

### 4.5 Correlation between age and PTC risk

In recent years, numerous clinical studies and meta-analyses have confirmed that the age distribution of PTC shows characteristic patterns, with differences in risk and tumor biological behavior across age groups. Our study used the age stratification according to the 8th edition of the American Joint Committee on Cancer (AJCC) classification, using 55 years as a cut-off. The results showed that age ≥55 years (OR = 0.318, 95%CI: 0.238–0.424, P<0.001) was an independent protective factor for PTC incidence. From an overall incidence trend perspective, the incidence of PTC is increasing among younger populations, with 61.5% of patients diagnosed under age 55, and most present with early-stage, low-risk disease (34). However, clinicopathological studies show that using 55 years as a cut-off, the malignancy of PTC in middle-aged and elderly patients (≥55 years) is significantly higher. Their risk of locally advanced disease (OR = 5.21) and lymph node metastasis (OR = 4.76) are both higher than in younger patients, and distant metastasis and disease-related death only occur in this group (35). Firstly, the reserve and stress-induced fluctuations of TSH in young and middle-aged individuals are greater than in the elderly (36). TSH is a major proliferative signal for thyroid follicular epithelial cells. It binds to TSH receptors (TSHR) on the cell membrane, activating downstream pathways such as PI3K/Akt and MAPK, promoting cell proliferation. Simultaneously, TSH can increase the susceptibility of thyroid cells to carcinogenic factors, induce DNA damage, and inhibit repair (37). A related meta-analysis indicated a significant relationship between higher TSH levels and the risk of differentiated TC (38). It should be emphasized that this “protective-like association” is only observed in our retrospective surgical cohort and cannot be extrapolated to the general population. This result stems from surgical referral-related selection bias: younger patients often undergo surgery for screen-detected small suspicious nodules, while elderly patients are primarily operated for large, symptomatic benign goiters or compressive lesions, resulting in a lower PTC proportion among older surgical candidates. Notably, this finding does not conflict with the established consensus that advanced age confers poorer tumor-specific prognosis after PTC develops.

### 4.6 Correlation between marital status and PTC risk

Marital status, as a sociodemographic factor, has become an important aspect of tumor epidemiology regarding its impact on malignancy risk. Univariate and multivariate logistic regression analyses in this study both showed a significant association between marital status and PTC risk. Specifically, compared with “unmarried” status, “married” status (OR = 0.260, P = 0.074) and “other” marital status (including divorced, widowed, etc., OR = 0.067, P = 0.003) were independent protective factors for PTC incidence. This finding suggests that individuals in married relationships have a significantly lower risk of developing PTC compared to unmarried individuals. Firstly, married individuals generally have more robust social networks, and health supervision and care from spouses can enhance awareness of disease screening (39). For example, married individuals may be more inclined to undergo regular health check-ups, facilitating early detection and monitoring of thyroid nodules; however, as this study included only patients who underwent surgery for thyroid nodules, this bias was controlled. They may also receive instrumental support from spouses (e.g., assistance with transportation and household tasks), allowing them to focus fully on treatment (40). Importantly, spouses can provide emotional support, which can alleviate the stress of cancer treatment (41). Studies have shown that the effect of social support provided by spouses may operate at the physiological level through neuroendocrine, neural, and immune interactions directly related to cancer (42, 43). Given the unclear causal interpretation and the borderline significance for married category, marital status was retained in the final nomogram only for exploratory purpose; we explicitly caution readers against over-interpreting marital status as a genuine causal protective factor for PTC. External validation in larger independent cohorts is required before drawing firm conclusions on marital status effect.Existing literature regarding marital status and PTC risk is scarce; social support, psychological stress, healthcare-seeking behavior are potential non-biological pathways, but cannot sufficiently explain the observed OR magnitude in our study.

### 4.7 Study limitations

First, this is a single-center retrospective study enrolling only surgical patients, which introduces referral and selection bias. Therefore, the findings cannot be generalized to patients with thyroid nodules who do not undergo surgery. Second, although temporal internal validation was performed, the prediction model has not been independently validated in populations from other medical centers. Its generalizability needs to be confirmed by future multicenter prospective studies. Imaging features, family history and radiation exposure history should be incorporated in future models to improve discriminatory performance. Finally, ABO blood-group status represents another readily-available host-related risk factor reported in previous literature; however, it was not accessible in our dataset and therefore could not be incorporated into the present nomogram. Large-scale prospective studies should be conducted in the future to include ABO blood-group status.

## 5 Conclusion

In conclusion, combined HT, combined MetS, and elevated UA are independent risk factors for PTC onset, whereas age ≥ 55 years, other marital status, and PNI level represent independent protective factors. Our research successfully established and verified a nomogram for predicting PTC risk in patients with thyroid nodules undergoing surgery, which may help clinicians identify high-risk candidates within this surgical population and facilitate individualized clinical decision-making.

## Data Availability

The original contributions presented in the study are included in the article/**Supplementary Material**. Further inquiries can be directed to the corresponding authors.

## Supplementary material

Supplementary Figure 1 The DCA curve of the risk nomogram for papillary thyroid carcinoma in the validation cohort.

## References

[1] Pizzato M Li M Vignat J Laversanne M Singh D La Vecchia C . The epidemiological landscape of thyroid cancer worldwide: GLOBOCAN estimates for incidence and mortality rates in 2020. Lancet Diabetes Endocrinol. (2022) 10:264–72. doi: 10.1016/s2213-8587(22)00035-3 35271818

[2] Sung H Ferlay J Siegel RL Laversanne M Soerjomataram I Jemal A . Global cancer statistics 2020: GLOBOCAN estimates of incidence and mortality worldwide for 36 cancers in 185 countries. CA Cancer J Clin. (2021) 71:209–49. doi: 10.3322/caac.21660 33538338

[3] Ito Y Miyauchi A Kihara M Fukushima M Higashiyama T Miya A . Overall survival of papillary thyroid carcinoma patients: A single-institution long-term follow-up of 5897 patients. World J Surg. (2018) 42:615–22. doi: 10.1007/s00268-018-4479-z 29349484 PMC5801380

[4] Lee E Jung W Woo JS Lee JB Shin BK Kim HK . Tumor sprouting in papillary thyroid carcinoma is correlated with lymph node metastasis and recurrence. Korean J Pathol. (2014) 48:117–25. doi: 10.4132/koreanjpathol.2014.48.2.117 24868224 PMC4026802

[5] Xing M . Molecular pathogenesis and mechanisms of thyroid cancer. Nat Rev Cancer. (2013) 13:184–99. doi: 10.1038/nrc3431 23429735 PMC3791171

[6] Hanahan D Weinberg RA . Hallmarks of cancer: The next generation. Cell. (2011) 144:646–74. doi: 10.1016/j.cell.2011.02.013 21376230

[7] Mantovani A Allavena P Sica A Balkwill F . Cancer-related inflammation. Nature. (2008) 454:436–44. doi: 10.1038/nature07205 18650914

[8] Arends J Bachmann P Baracos V Barthelemy N Bertz H Bozzetti F . ESPEN guidelines on nutrition in cancer patients. Clin Nutr. (2017) 36:11–48. doi: 10.1016/j.clnu.2016.07.015 27637832

[9] Aydoğdu YF BüyükKasap Ç Göbüt H Akin M . ABO blood group and Rhesus factor as potential markers in papillary thyroid cancer: A retrospective comparative analysis (2015–2023). Langenbecks Arch Surg. (2025) 411:25. doi: 10.1007/s00423-025-03920-4 41313453 PMC12662903

[10] Bliddal S Nielsen CH Feldt-Rasmussen U . Recent advances in understanding autoimmune thyroid disease: The tallest tree in the forest of polyautoimmunity. F1000Res. (2017) 6:1776. doi: 10.12688/f1000research.11535.1 29043075 PMC5621109

[11] Ralli M Angeletti D Fiore M D'Aguanno V Lambiase A Artico M . Hashimoto's thyroiditis: An update on pathogenic mechanisms, diagnostic protocols, therapeutic strategies, and potential malignant transformation. Autoimmun Rev. (2020) 19:102649. doi: 10.1016/j.autrev.2020.102649 32805423

[12] Dailey ME Lindsay S Skahen R . Relation of thyroid neoplasms to Hashimoto disease of the thyroid gland. AMA Arch Surg. (1955) 70:291–7. doi: 10.1001/archsurg.1955.01270080137023 13227748

[13] Greten FR Grivennikov SI . Inflammation and cancer: Triggers, mechanisms, and consequences. Immunity. (2019) 51:27–41. doi: 10.1016/j.immuni.2019.06.025 31315034 PMC6831096

[14] Turizo-Smith AD Córdoba-Hernandez S Mejía-Guarnizo LV Monroy-Camacho PS Rodríguez-García JA . Inflammation and cancer: Friend or foe? Front Pharmacol. (2024) 15:1385479. doi: 10.3389/fphar.2024.1385479 38799159 PMC11117078

[15] Malhab LJB Saber-Ayad MM Al-Hakm R Nair VA Paliogiannis P Pintus G . Chronic inflammation and cancer: The role of endothelial dysfunction and vascular inflammation. Curr Pharm Des. (2021) 27:2156–69. doi: 10.2174/1381612827666210303143442 33655853

[16] Abbasgholizadeh P Naseri A Nasiri E Sadra V . Is Hashimoto thyroiditis associated with increasing risk of thyroid malignancies? A systematic review and meta-analysis. Thyroid Res. (2021) 14:26. doi: 10.1186/s13044-021-00117-x 34861884 PMC8641157

[17] Yang Y Liu J Shi X Wang M . Clinical and pathological characteristics of patients with papillary thyroid carcinoma coexisting with Hashimoto's thyroiditis: A retrospective cohort study. Cancer Control. (2023) 30:10732748231199647. doi: 10.1177/10732748231199647 37643366 PMC10467246

[18] Xie Y Xu P Liu K Lin S Wang M Tian T . Hyperuricemia and gout are associated with cancer incidence and mortality: A meta-analysis based on cohort studies. J Cell Physiol. (2019) 234:14364–76. doi: 10.1002/jcp.28138 30693505 PMC13482836

[19] Ruban M Pozhidaeva E Bolotina L Kaprin A . The role of diet and nutrition in cancer development and management: From molecular mechanisms to personalized interventions. Foods. (2025) 14:1788. doi: 10.3390/foods14101788 40428567 PMC12110988

[20] Qu Z Lu YJ Feng JW Chen YX Shi LQ Chen J . Preoperative prognostic nutritional index and neutrophil-to-lymphocyte ratio predict survival outcomes of patients with hepatocellular carcinoma after curative resection. Front Oncol. (2021) 11:823054. doi: 10.3389/fonc.2021.823054 PMC8831760 35155212

[21] Li J Zhu N Wang C You L Guo W Yuan Z . Preoperative albumin-to-globulin ratio and prognostic nutritional index predict the prognosis of colorectal cancer: A retrospective study. Sci Rep. (2023) 13:17272. doi: 10.1038/s41598-023-43391-5 37828259 PMC10570287

[22] Karra P Winn M Pauleck S Bulsiewicz-Jacobsen A Peterson L Coletta A . Metabolic dysfunction and obesity-related cancer: Beyond obesity and metabolic syndrome. Obes (Silver Spring). (2022) 30:1323–34. doi: 10.1002/oby.23444 35785479 PMC9302704

[23] Huang PL . A comprehensive definition for metabolic syndrome. Dis Model Mech. (2009) 2:231–7. doi: 10.1242/dmm.001180 19407331 PMC2675814

[24] Park JH Choi M Kim JH Kim J Han K Kim B . Metabolic syndrome and the risk of thyroid cancer: A nationwide population-based cohort study. Thyroid. (2020) 30:1496–504. doi: 10.1089/thy.2019.0699 32524894

[25] Kim J Han K Kim MK Baek KH Song KH Kwon HS . Cumulative exposure to metabolic syndrome increases thyroid cancer risk in young adults: A population-based cohort study. Korean J Intern Med. (2023) 38:526–33. doi: 10.3904/kjim.2022.405 37198877 PMC10338240

[26] Sun Y Lin X Zou Z Zhou Y Liu A Li X . Association between visceral fat area and metabolic syndrome in individuals with normal body weight: Insights from a Chinese health screening dataset. Lipids Health Dis. (2025) 24:57. doi: 10.1186/s12944-025-02482-0 39966964 PMC11837645

[27] Ramos CF Zamoner A . Thyroid hormone and leptin in the testis. Front Endocrinol (Lausanne). (2014) 5:198. doi: 10.3389/fendo.2014.00198 25505448 PMC4243692

[28] Blandin A Amosse J Froger J Hilairet G Durcin M Fizanne L . Extracellular vesicles are carriers of adiponectin with insulin-sensitizing and anti-inflammatory properties. Cell Rep. (2023) 42:112866. doi: 10.1016/j.celrep.2023.112866 37605533

[29] Li Z Su Y Su H Pan J Li S Lu L . Serum uric acid and its metabolism-a vital factor in the inflammatory transformation of cancer. J Adv Res. (2025) 12(2):1685–708. doi: 10.1016/j.jare.2025.08.031 PMC13131520 40850676

[30] Fan K Sun T Yin F . J-shaped association between uric acid and breast cancer risk: A prospective case-control study. J Cancer Res Clin Oncol. (2023) 149:7629–36. doi: 10.1007/s00432-023-04725-y 36995406 PMC10374747

[31] So A Thorens B . Uric acid transport and disease. J Clin Invest. (2010) 120:1791–9. doi: 10.1172/jci42344 20516647 PMC2877959

[32] Braga TT Forni MF Correa-Costa M Ramos RN Barbuto JA Branco P . Soluble uric acid activates the NLRP3 inflammasome. Sci Rep. (2017) 7:39884. doi: 10.1038/srep39884 28084303 PMC5233987

[33] Crišan TO Cleophas MC Oosting M Lemmers H Toenhake-Dijkstra H Netea MG . Soluble uric acid primes TLR-induced proinflammatory cytokine production by human primary cells via inhibition of IL-1Ra. Ann Rheum Dis. (2016) 75:755–62. doi: 10.1136/annrheumdis-2014-206564 25649144

[34] Li C Hsieh MC Chang SJ . Metabolic syndrome, diabetes, and hyperuricemia. Curr Opin Rheumatol. (2013) 25:210–6. doi: 10.1097/bor.0b013e32835d951e 23370374

[35] Kim SJ Durr ML Darbinian JA Sakoda LC Meltzer CJ Arzumanyan H . Ten-year thyroid cancer incidence in an integrated healthcare delivery system. Perm J. (2021) 25:20.209. doi: 10.7812/tpp/20.209 33970070 PMC8817903

[36] Kaliszewski K Diakowska D Nowak Ł Wojtczak B Rudnicki J . The age threshold of the 8th edition AJCC classification is useful for indicating patients with aggressive papillary thyroid cancer in clinical practice. BMC Cancer. (2020) 20:1166. doi: 10.1186/s12885-020-07636-0 33256657 PMC7708212

[37] Bi WK Xu H Tian ZH Teng W Zheng GW Yin QQ . Stimulated thyrotropin (TSH) levels were inversely correlated with age. Int J Gen Med. (2024) 17:6479–86. doi: 10.2147/ijgm.s497208 39742031 PMC11687293

[38] Xu B Gu SY Zhou NM Jiang JJ . Association between thyroid stimulating hormone levels and papillary thyroid cancer risk: A meta-analysis. Open Life Sci. (2023) 18:20220671. doi: 10.1515/biol-2022-0671 37588997 PMC10426723

[39] Su A Zhao W Wu W Wei T Ruan M Li Z . The association of preoperative thyroid-stimulating hormone level and the risk of differentiated thyroid cancer in patients with thyroid nodules: A systematic review and meta-analysis. Am J Surg. (2020) 220:634–41. doi: 10.1016/j.amjsurg.2020.01.009 31955813

[40] Krajc K Miroševič Š Sajovic J Klemenc Ketiš Z Spiegel D Drevenšek G . Marital status and survival in cancer patients: A systematic review and meta-analysis. Cancer Med. (2023) 12:1685–708. doi: 10.1002/cam4.5003 PMC9883406 35789072

[41] Mihor A Tomsic S Zagar T Lokar K Zadnik V . Socioeconomic inequalities in cancer incidence in Europe: A comprehensive review of population-based epidemiological studies. Radiol Oncol. (2020) 54:1–13. doi: 10.2478/raon-2020-0008 32074075 PMC7087422

[42] de Graeff A de Leeuw JR Ros WJ Hordijk GJ Blijham GH Winnubst JA . Sociodemographic factors and quality of life as prognostic indicators in head and neck cancer. Eur J Cancer. (2001) 37:332–9. doi: 10.1016/s0959-8049(00)00385-3 11239754

[43] Huang TB Zhou GC Dong CP Wang LP Luan Y Ye JT . Marital status independently predicts prostate cancer survival in men who underwent radical prostatectomy: An analysis of 95,846 individuals. Oncol Lett. (2018) 15:4737–44. doi: 10.3892/ol.2018.7964 29552113 PMC5840566

